# Parkinson’s Disease Detection Using Filter Feature Selection and a Genetic Algorithm with Ensemble Learning

**DOI:** 10.3390/diagnostics13172816

**Published:** 2023-08-31

**Authors:** Abdullah Marish Ali, Farsana Salim, Faisal Saeed

**Affiliations:** 1 Department of Computer Science, Faculty of Computing and Information Technology, King Abdulaziz University, Jeddah 21589, Saudi Arabia; ammali@kau.edu.sa; 2 DAAI Research Group, College of Computing and Digital Technology, Birmingham City University, Birmingham B4 7XG, UK; farsana.salim@bcu.ac.uk

**Keywords:** Parkinson’s disease (PD), filter feature selection, ensemble learning, genetic selection

## Abstract

Parkinson’s disease (PD) is a neurodegenerative disorder marked by motor and non-motor symptoms that have a severe impact on the quality of life of the affected individuals. This study explores the effect of filter feature selection, followed by ensemble learning methods and genetic selection, on the detection of PD patients from attributes extracted from voice clips from both PD patients and healthy patients. Two distinct datasets were employed in this study. Filter feature selection was carried out by eliminating quasi-constant features. Several classification models were then tested on the filtered data. Decision tree, random forest, and XGBoost classifiers produced remarkable results, especially on Dataset 1, where 100% accuracy was achieved by decision tree and random forest. Ensemble learning methods (voting, stacking, and bagging) were then applied to the best-performing models to see whether the results could be enhanced further. Additionally, genetic selection was applied to the filtered data and evaluated using several classification models for their accuracy and precision. It was found that in most cases, the predictions for PD patients showed more precision than those for healthy individuals. The overall performance was also better on Dataset 1 than on Dataset 2, which had a greater number of features.

## 1. Introduction

Parkinson’s disease (PD) is a neurodegenerative disorder that affects millions of individuals worldwide. It is characterized by motor symptoms such as tremors, rigidity, bradykinesia (slowness of movement), and postural instability. PD not only impairs the quality of life for patients but also poses significant challenges for accurate and timely diagnosis. The presence of voice deficits, which are frequently defined by alterations in speech patterns, cadence, and tone, emerges as an important element of Parkinson’s disease symptomatology. The study by Tjaden, Lam, and Wilding [1] revealed that speakers with PD displayed expanded peripheral and non-peripheral vowel space areas during articulate speech, accompanied by a reduction in speech rate and an increased vocal intensity. Furthermore, the study by Tsanas et al. [2] highlighted the feasibility of utilizing straightforward, self-administered, and non-intrusive speech tests as a potential strategy for regular, remote, and precise monitoring of PD symptom progression with the employment of the Unified Parkinson’s Disease Rating Scale (UPDRS). These studies showcase the potential of voice-related changes to act as valuable indicators for the early detection of Parkinson’s disease, despite receiving less recognition than motor symptoms.

Recent advances in machine learning techniques, as well as the availability of large-scale datasets, have opened new avenues for the automated identification of PD utilizing various forms of data, including voice recordings. Furthermore, machine learning-based PD detection systems have the potential to be non-invasive, low-cost, and easily scalable. A voice recording can be collected easily through commonly available devices such as smartphones, making it a convenient and accessible tool for screening and monitoring PD. 

A series of studies have delved into the domain of Parkinson’s disease (PD) classification, harnessing voice data as a diagnostic indicator. However, one notable gap is the limited size and diversity of the datasets employed in many prior studies. This limitation raises concerns about the generalizability and reliability of the resulting classification models. This study makes a significant contribution to the field by decisively addressing this issue through the utilization of two distinct datasets. Another gap has been the lack of comprehensive feature selection methods employed in PD classification studies. While some efforts have been made to apply feature selection techniques, this study takes a step forward by introducing a novel combination of filter feature selection methods with ensemble learning and genetic selection. This fusion holds the promise of uncovering more relevant and discriminative features inherent in the voice data, potentially leading to a substantial enhancement in the accuracy of PD classification. Furthermore, the limited exploration of model ensemble techniques in prior studies has presented a significant gap, which this research effectively addresses. While several investigations have focused primarily on individual classification algorithms, the untapped potential of leveraging the strengths of various algorithms through ensemble methods has been underutilized. Ensemble learning methods have the inherent advantage of integrating the diverse strengths of different algorithms, thereby enhancing the overall predictive power and accuracy of the classification process. By exploring this avenue, this research provides a vital contribution to the field by demonstrating the potential of ensemble techniques to significantly elevate the performance and efficacy of PD classification models.

In this study, a combination of filter feature selection methods with ensemble learning and genetic selection was used to detect PD from voice clips. The filtered data was fed into different classification models, which were then evaluated based on their accuracy and precision. By evaluating the models on these diverse datasets with varying characteristics and complexities, the generalizability and scalability of the approach may be assessed. The outcomes of this study may enhance our understanding and augment the efficacy of early PD detection, ultimately leading to improved patient care and prognosis.

## 2. Related Work

Several studies have investigated the use of machine learning and statistical modelling techniques to extract discriminative features from voice recordings and to develop classification models for PD detection. These have been summarized in Table 1.

In a recent study by Sheikhi and Kheirabadi [3], a voice dataset from the UCI Repository was utilized for the classification of PD. The dataset comprised voice recordings from 42 patients, totaling 5875 instances. They proposed a model that combined the Random Forest (RF) and Rotation Forest algorithms to classify the predictions into two categories: severe or non-severe. The accuracy results for the total Unified PD Rating Scale (UPDRS) and motor UPDRS using this model were found to be 76.09% and 79.49%, respectively. In another study conducted by Mohammed et al. [4], a multi-agent approach was employed to filter and identify the most relevant features that could enhance PD classification accuracy while reducing training time. They utilized a dataset consisting of 31 human voice recordings, 23 of which were diagnosed with PD. Initially, the dataset contained 22 features, which were reduced to 14 after the filtering process. Eleven different classification algorithms were applied to the selected features, and the results were evaluated. This approach achieved an accuracy of 96.6%.

Recently, Velmurugan and Dhinakaran [5] proposed an approach known as the Ensemble Stacking Learning Algorithm (ESLA) for PD classification. The ESLA method integrated the linear regression and Adaboost ensemble techniques with the RF and Extreme Gradient Boosting (XGBoost) algorithms to effectively identify individuals with PD. The dataset employed was collected from 188 PD patients. Initially, basic models were developed using the RF and XGBoost algorithms for prediction. Subsequently, the outputs of these prediction models were utilized as inputs in the next step to fine-tune parameters and create models with enhanced accuracy. The top models for the RF and XGBoost were then chosen. The RF model’s accuracy increased from 84.21% to 84.86%, while the XGBoost model’s accuracy increased from 88.15% to 88.85%. The proposed ESLA method leveraged the stacking technique to create four stacked models, combining RF, XGBoost, logistic regression, Adaboost, and multilayer perceptron (MLP), to further enhance the classification performance. This method outperformed the individual classifiers, yielding an accuracy of 90.13%.

The study by Sharma et al. [6] proposed a binary version of the Rao algorithm to overcome the problem of feature selection. The Rao algorithm was applied to four public PD datasets using the kNN classifier for PD classification. The highest accuracy of the classifications obtained from the four datasets was 99.25%. 

In their study, Sabeena et al. [7] proposed a novel framework for feature selection and classification to identify individuals with PD. The dataset used consisted of speech samples from 188 PD patients and 64 healthy individuals. An optimization-based ensemble feature selection method was employed. It involved three different approaches for selecting the optimized subsets of features. The results from these approaches were combined using an ensemble technique. The selected features were then utilized in various classifiers, which yielded accuracies ranging from 83.66% to 98.77%. In another study by Ul Haq et al. [8], a dataset of 196 voice samples with 23 attributes was utilized. Among the 31 individuals in the dataset, 23 were diagnosed with PD, and eight were considered healthy. Relief-ant-colony optimization (ACO), and Relief-ACO methods were employed to select subsets of features. The selected feature subsets were then used with the SVM classifier. The results showed that when the Relief-ACO feature selection method was combined with SVM using the radial basis function (RBF) kernel, an accuracy of 98.20% was achieved, outperforming other feature selection methods. Similarly, when used with SVM using the linear kernel, the Relief-ACO feature selection method achieved a high accuracy of 99.50% compared to other feature selection methods.

In the study conducted by Sarankumar et al. [9], a dataset of voice data collected from 42 patients was analyzed. The dataset contained a total of 5875 audio files. After preprocessing the dataset, a clustering process was performed using wavelet cleft fuzzy. Next, feature selection was carried out from the clustering step using the firming bacteria foraging algorithm. The selected features were then employed to predict PD patients using the Deep Brooke inception net classification algorithm, resulting in an accuracy of 99.88%. In another study by Pahuja and Nagabhushan [10], a free voice dataset of PD patients from the UCI repository was used. This dataset had six recordings for each patient. Classification algorithms ANN, SVM, and kNN, were employed and achieved accuracies of 95.89%, 88.21%, and 72.31%, respectively.

The research conducted by Yücelbaş [11] used a dataset comprising voice recordings of 252 individuals. The dataset employed 188 patients with PD and 64 healthy individuals, with three recordings for each person, resulting in a total of 756 recordings. The study proposed an information gain algorithm-based KNN hybrid model (IGKNN) for feature selection analysis. The proposed IGKNN method, using 22 selected features, achieved an accuracy of 98%. Pramanik et al. [12] used a publicly available dataset from the UCI machine learning repository in a different study. This dataset included 752 acoustic features for 252 people, including 188 PD patients and 64 healthy people. A total of 21 baseline features (BF), 22 vocal fold features (VFF), and 11-time frequency features (TFF) were extracted from this dataset. A collaborative feature bank was built to evaluate the performance of PD detection using three feature selection techniques: Correlated Feature Selection (CFS), Fisher Score Feature Selection (FSFS), and Mutual Information-based Feature Selection (MIFS). The Naïve Bayes classifier was used in the evaluation. The best accuracy obtained from utilizing the three feature selection strategies was 78.97%.

The study conducted by Salmanpour, Shamsaei, Saberi, et al. [13] aimed to categorize PD into its distinct subtypes. To achieve this, the researchers compiled 30 datasets over a period of four years from 885 individuals diagnosed with Parkinson’s Progressive Marker and 163 healthy individuals. These datasets encompassed a combination of non-imaging, imaging, and radiomic features extracted from DAT-SPECT images. The study used 16 algorithms for feature reduction, eight algorithms for clustering, and 16 classifiers. The radiomics features aided in generating a consistent cluster structure, enabling the subdivision of PD into three distinct subtypes: mild, intermediate, and severe.

The study by Nahar et al. [14] was based on 44 acoustic features extracted from a dataset of 80 people, 40 of whom were PD patients and 40 who were healthy. The feature selection was performed using three different methods: Boruta, Recursive Feature Elimination (RFE), and RF. Gradient Boosting, Extreme Gradient Boosting, Bagging, and an Extra Tree Classifier were employed. The classifier results were examined using the original 44 features, and the Extreme Gradient Boosting classifier achieved a good accuracy of 78.08%. Furthermore, the classification results were analyzed after using the three feature selection methods, and an accuracy of 82.35% was achieved using the RFE feature selection method and the Bagging classifier.

While previous studies have explored PD diagnosis using voice analysis, significant gaps remain. Concerns related to generalizability and accuracy have been highlighted due to the inadequate dataset diversity and feature selection methodologies. This study tackles these limitations by combining two independent datasets and offering a fusion of filter feature selection with ensemble learning and genetic selection.

## 3. Materials and Methods

### 3.1. Datasets

Two distinct biomedical voice datasets were employed in this study for the assessment of PD. 

The first dataset encompasses a compilation of biomedical voice measurements obtained from 31 individuals, 23 of whom were diagnosed with PD. Each row in the dataset corresponds to a voice recording from these individuals, while each column represents a specific voice measure. This dataset was expertly curated through a collaborative effort between Max Little of the University of Oxford and the National Center for Voice and Speech in Denver, Colorado, entailing the meticulous recording of speech signals [15]. 

The dataset contains 195 sustained vowel phonations, encompassing a range of time since diagnosis spanning from 0 to 28 years. The subjects’ ages vary from 46 to 85 years, with a mean age of 65.8 and a standard deviation of 9.8. For each subject, an average of six phonations were captured, varying in duration from one to 36 s. These phonations were recorded within an IAC sound-treated booth, utilizing a head-mounted microphone (AKG C420) positioned 8 cm away from the lips. The calibration of the microphone involved a Class 1 sound level meter (B&K 2238) situated 30 cm from the speaker. The voice signals were directly recorded onto a computer through CSL 4300B hardware (Kay Elemetrics), sampled at 44.1 kHz, and with a 16 bit resolution. To ensure the robustness of the algorithms, all samples underwent digital amplitude normalization prior to the computation of the metrics. The details of the subjects are given in Table 2.

The second dataset utilized in this study was built by Sakar et al. [16] for their study, which comprised a comparative analysis of speech signal processing algorithms for PD classification and the use of the tunable Q-factor wavelet transform. This dataset was collected at the Department of Neurology in the Cerrahpaşa Faculty of Medicine, Istanbul University. It entailed the comprehensive data of 188 PD patients (107 men and 81 women) spanning an age range of 33 to 87 years (mean age: 65.1 ± 10.9). Additionally, a control group consisting of 64 healthy individuals (23 men and 41 women) with ages ranging from 41 to 82 years (mean age: 61.1 ± 8.9) was included. During the data collection process, voice recordings were captured using a microphone set to a frequency of 44.1 KHz. Specifically, sustained phonation of the vowel /a/ was necessary to collect from each subject with three repetitions. Subsequently, a comprehensive set of speech signal processing algorithms, including Time-Frequency features, Mel Frequency Cepstral Coefficients (MFCCs), Wavelet Transform-based features, Vocal Fold features, and TWQT features, were diligently applied to the speech recordings of PD patients.

### 3.2. Filter Feature Selection

The goal of feature selection in machine learning and data mining is to identify and maintain a subset of important features from the original dataset. The motivation for feature selection stems from its ability to increase model performance, reduce computational complexity, and improve model interpretability. Filter methods have received substantial attention among the various approaches to feature selection due to their simplicity, efficiency, and capacity to evaluate feature significance independently of any specific learning algorithm.

Filter feature selection methods attempt to prioritize and choose features based on their unique properties and association with the target variable without considering the learning process of the specific model. In this study, we focus on the importance of filtering quasi-constant features as a crucial step in the filter feature selection process. Quasi-constant features refer to those with minimal variance or almost constant values across the dataset, providing limited or negligible discriminatory information. 

Identifying and removing quasi-constant features reduces dimensionality and improves model generalization. By eliminating these features, we may reduce noise, improve computational performance, and promote more meaningful dataset exploration. However, to effectively filter out quasi-constant features, it is essential to set an appropriate threshold that determines the acceptable level of variance below which a feature is considered quasi-constant and subsequently removed.

### 3.3. Genetic Algorithm

Genetic Algorithms (GAs), members of the evolutionary algorithm family, have emerged as a popular and robust solution to addressing the limitations encountered by conventional optimization techniques in terms of efficiency and effectiveness. They are inspired by concepts of natural selection and genetics, imitating the process of evolution to find optimal solutions within a specific area.

The concept of a population-based search is at the heart of GAs, in which a set of potential solutions, referred to as individuals or chromosomes, undergo iterative refinement to explore the solution space. GAs enable the propagation of desirable features and the examination of new solution regions by utilizing genetic operators such as selection, crossover, and mutation. This population-centric method enables GAs to tackle complicated optimization problems with high dimensionality, non-linearity, and multimodality effectively.

GAs work by iteratively generating new populations, with each population being evaluated based on a fitness function that assesses the quality of individual solutions. They promote convergence towards optimal or near-optimal solutions across generations by repeatedly applying selection, crossover, and mutation operators. This repeated exploration and exploitation approach enables them to navigate the solution space with ease, exceeding local optima and delivering strong solutions.

### 3.4. Methods

Two distinct methods were employed in the experiments to evaluate the effectiveness of the filtering approach. 

The selection of the quasi-constant threshold for filtering features was performed using a trial-and-error method. After careful evaluation of different threshold values, it was found that the best results were achieved when the threshold was set to 0.0001. However, both lower and higher threshold values yielded decreased accuracy in our experiments.

A combination of filter feature selection and ensemble learning methods was employed in the first method. First, quasi-constant features with a threshold value of 0.0001 were identified and subsequently removed from the dataset, resulting in a refined dataset. This refined dataset was then subjected to five different classification algorithms: Gaussian Naïve Bayes classifier, Support Vector Machine (SVM), Decision Tree, Random Forest, and XGBoost.

**Figure 1:**
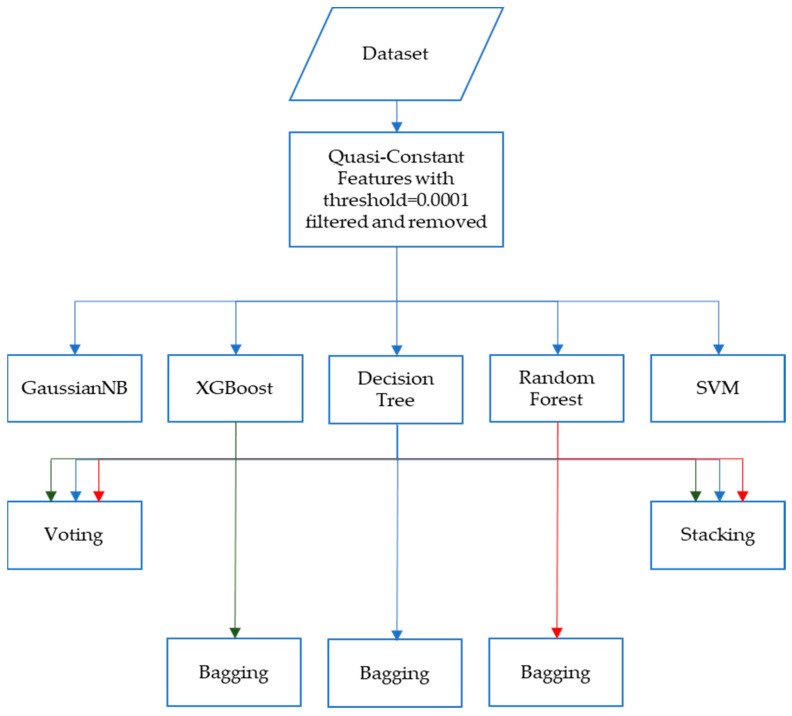
Method 1, where filter feature selection was applied to five different classification algorithms followed by ensemble learning methods.

The performance evaluation revealed that among the tested classification algorithms, Decision Tree, Random Forest, and XGBoost exhibited the highest classification accuracy and predictive power. Building upon this finding, further analysis was conducted by employing ensemble learning methods: stacking and voting, using the three best-performing algorithms. Additionally, bagging was also applied to the three selected algorithms to explore potential performance enhancements and model robustness. The first method is summarized in Figure 1.

**Figure 2:**
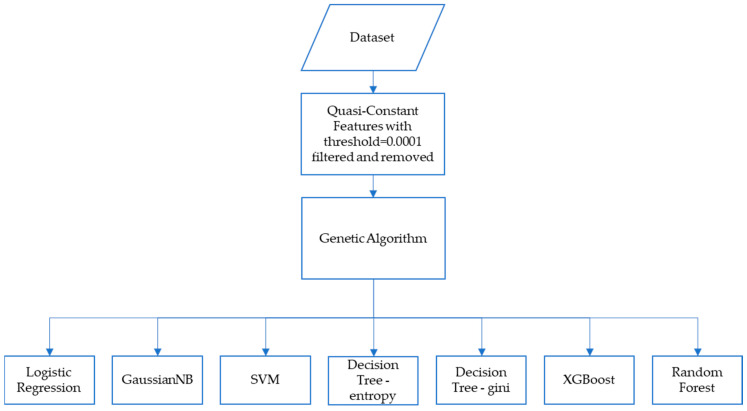
Method 2, where filter feature selection is followed by a genetic algorithm before applying to the classification models.

In the second method, after filtering out the quasi-constant features, a genetic algorithm was utilized to further optimize the feature selection process for the same set of classification algorithms: GaussianNB, SVM, Decision Tree (with entropy and Gini index), XGBoost, Random Forest, and additionally, logistic regression. A pictorial representation of the second method is shown in Figure 2.

The genetic selection was performed after 40 generations of populations with 50 individuals. The crossover probability was 0.5, and the mutation probability was 0.2. The crossover independent probability was set to 0.5 and the mutation independent probability to 0.05. The tournament size was set to three, and the number of generations after which the optimization is terminated when the best individual has not changed in all the previous generations (n_gen_no_change) was set to 10.

## 4. Results and Discussion

With the quasi-constant threshold set to 0.0001, five of the twenty-four features in Dataset 1 and 188 of the 754 features in Dataset 2 were identified as quasi-constant and subsequently eliminated. This was a significant number of features in both datasets and enabled streamlining the feature space to enhance the efficiency and effectiveness of subsequent modeling tasks.

Different classification models were then tested on the filtered datasets. The accuracies of these models are given in Table 3. 

Among the various models tested, Decision Tree, Random Forest, and XGBoost demonstrated notably higher accuracies compared to the others on both datasets. Therefore, ensemble methods, namely voting, stacking, and bagging, were applied to these three models. Both hard voting and soft voting were employed. The models were stacked to leverage the strengths of multiple classifiers. Bagging was applied independently to each of the three models, utilizing 5-fold cross-validation and a total of 500 trees. The resulting accuracies obtained from these ensemble approaches on Dataset 1 and Dataset 2 are presented in Table 4 and Table 5, respectively.

Voting with both hard and soft voting classifiers attained perfect accuracy (100%) on Dataset 1. Stacking also displayed good results, with a 96.2% accuracy on Dataset 1 and a 90.06% accuracy on Dataset 2. Bagging had lower accuracy than voting and stacking.

Ensemble approaches take advantage of the diversity and complementary features of individual models, resulting in higher accuracy. The perfect accuracy achieved by voting on Dataset 1 suggests strong agreement among the models, contributing to accurate classification. The relatively high accuracy of the stacked models verifies the efficiency of combining the predictions of the base models to produce greater performance. Bagging decreases the variance and instability of classification models by training individual models on diverse subsets of the dataset and aggregating their predictions. The slight decrease in accuracy from bagging could have resulted from the intrinsic randomness introduced during the resampling process, which may result in a minor trade-off between accuracy and model stability.

In the second method, the filtered dataset was subjected to further feature refinement using genetic selection. A genetic algorithm investigates several feature combinations to determine an optimal subset that achieves the maximum classification accuracy. The genetic selection process begins with the generation of an initial population of potential feature subsets, each of which represents a unique combination of features. These subsets were then analyzed using the same classification models in addition to logistic regression. The results thus obtained are summarized in Table 6.

It can be observed that the accuracy of the Guassian Naïve Bayes classifier improved to 91.83% for Dataset 1 and 77.63% for Dataset 2 after genetic selection. This indicates that genetic algorithms were effective in choosing the relevant features that boosted the performance of the classifier. However, with the SVM classifier, the accuracy declined to 81.63% for Dataset 1 and improved only slightly for Dataset 2 with 77.63% accuracy. The accuracy of the decision tree model, measured using both entropy and the Gini index, also failed to improve significantly with genetic selection. The same was true for random forest and XGBoost classifiers. Logistic regression also produced similar results to the rest, with an accuracy of 89.79% with Dataset 1 and 76.97 with Dataset 2. In summary, genetic selection had varying effects on the accuracy of the different classification models. This implies that the effectiveness of genetic algorithms may also be dependent on the properties of the classification model.

Precision may also be an important evaluation metric for the detection of PD. Precision is a performance metric that quantifies the accuracy of a classification model’s positive predictions. It determines the proportion of true positive predictions (positive instances correctly identified) out of all predicted positive instances (true positives + false positives). By focusing on precision, we can ensure that the models accurately identify actual PD patients while also reducing the risks of misclassifying individuals in good health as having the disease, as that can lead to unnecessary fear, stress, and even medical interventions. A high precision score gives reliability and greater confidence to employ the models in PD diagnosis. The precision of the predictions made by the models with filter feature selection and genetic selections on both datasets is given in Table 7 and Table 8, respectively.

It is notable that the decision tree and XGBoost classifiers achieved perfect precision in identifying both PD patients and healthy individuals. The Gaussian Naïve Bayes and random forest classifiers attained perfect precision in identifying PD patients in Dataset 1, whereas the SVM classifier showed perfect precision in detecting healthy individuals in both datasets. It is also noteworthy that SVM was the only classifier that achieved perfect precision in identifying at least one category (PD patients or healthy individuals) in Dataset 2.

After genetic selection on Dataset 1, all the models achieved relatively high precision in identifying PD patients, ranging from 84% to 94%. The Gaussian Naïve Bayes classifier was 100% precise in identifying healthy individuals. However, all the other models showed less precision in identifying healthy individuals (ranging from 62% to 80%) than PD patients. The same can also be observed in the case of Dataset 2 after genetic selection. All models showed higher precision in identifying PD patients (ranging from 74% to 79%) than in identifying healthy individuals (ranging from 0% to 78%). This suggests that identifying PD patients may be easier than identifying healthy people from the selected datasets. One possible reason for this could be the unequal distribution of PD patients and healthy individuals in both datasets. Both datasets contained information from a higher number of PD patients than healthy people, which made the models more proficient in learning the patterns and characteristics associated with PD. This imbalance in class distribution may have led to a bias towards PD patients during the training process, potentially resulting in higher precision in identifying PD cases.

The overall results for Dataset 1 were better than those for Dataset 2. This disparity may be due to the difference in the number of features between the two datasets. Initially, Dataset 1 had only 24 features, which is substantially fewer than Dataset 2, which had 754 features. Even after applying the filter feature selection technique, a relatively large number of features (566 features) were preserved in Dataset 2 compared to Dataset 1. The presence of a larger feature space in Dataset 2 might have introduced additional complexity and made it more challenging for the models to discern the meaningful patterns associated with PD. This demonstrates that having a greater number of features may not necessarily translate to better results and may even generate noise or redundancy, resulting in poor model performance.

While previous research has established the efficacy of ensemble techniques [5,7], a comparative analysis with the current literature demonstrates a remarkable outperformance of ensemble learning methods, as exemplified by the perfect accuracy (100%) achieved by both hard and soft voting on Dataset 1. Moreover, the hard voting classifier achieved an accuracy of 91.53% on Dataset 2, surpassing the performance reported in the related literature [5]. The introduction of genetic selection is a novel approach. While certain models responded differently to genetic selection, this nuanced approach illustrates the complicated interplay between feature selection strategies and classification outcomes. Following genetic selection, the GaussianNB classifier achieved the best accuracy for both datasets, with an accuracy of 91.83% for Dataset 1 and 77.63% for Dataset 2. The emphasis on precision ensures that PD patients are accurately identified while minimizing the risk of misclassifying healthy individuals, a crucial aspect for real-world clinical applications. Filter feature selection led to perfect (100%) precision in the predictions of decision trees and XGBoost classifiers. With genetic selection, there was an average precision of 88.42% in identifying PD patients and 72% in identifying healthy individuals in Dataset 1. In Dataset 2, these values were 77.14% for PD patients and 55.43% for healthy individuals. This holistic viewpoint illustrates the depth and breadth of this research, effectively establishing its relevance and impact on improving patient care and prognosis. Overall, this research not only benchmarks favorably against the prior literature but also offers a novel strategy for enhancing the accuracy and reliability of PD detection through voice data analysis.

## 5. Conclusions and Future Scope

This study aimed to develop an efficient method for the detection of PD from voice clips. A combination of filter feature selection, ensemble learning, and genetic selection was employed. The results of the study demonstrated the effectiveness of filter feature selection in streamlining the feature space and enhancing the efficiency of subsequent modeling tasks. By eliminating quasi-constant features, a significant number of irrelevant features were successfully removed, leading to high model accuracy. The application of ensemble learning techniques, such as voting, stacking, and bagging, further explored the classification performance of these models. Additionally, the genetic selection approach analyzed the precision of the classification models in identifying PD patients and healthy individuals. The models exhibited relatively high precision in identifying PD patients, while the precision in identifying healthy individuals was comparatively lower. Moreover, the comparison between Dataset 1 and Dataset 2 demonstrated the effect of feature space on model performance. Dataset 1, with a smaller number of features, yielded better results compared to Dataset 2, which had a larger feature space even after filter feature selection.

While this study contributes significantly to the field of PD detection, a few limitations warrant careful consideration. The precision analysis performed in this study reveals a potential bias toward recognizing PD patients more accurately than healthy people. This bias stems from the inherent class imbalance within the datasets, where PD patients are overrepresented compared to healthy individuals. This discrepancy could lead to a skewed learning process, affecting the models’ generalizability when applied to larger, more balanced populations. Furthermore, the variation in performance between Dataset 1 and Dataset 2 underscores the sensitivity of model outputs to the dimensionality of the feature space. The larger feature set of Dataset 2, even after filter feature selection, suggests the possibility of increased noise or redundancy, thereby affecting model robustness and performance.

Future studies could explore the use of sampling techniques, such as oversampling or undersampling, to balance the datasets. This would help in achieving better performance and addressing the bias towards the majority class. The current study utilized specific datasets for model development and evaluation. Future research could involve testing the developed models on external datasets or real-world data to assess their generalizability and robustness. This would provide insights into the practical applicability of the proposed methods and their performance across different populations. By addressing these future research areas, we can further advance the field of PD detection from voice data and contribute to the development of accurate, reliable, and clinically applicable diagnostic tools.

## Figures and Tables

**Table 1:** Summary of studies that utilized machine learning for PD detection.

Study	Dataset	Method	Results
Sheikhi and Kheirabadi, 2022 [3]	Voice UCI PD dataset	Combination of the Random Forest (RF) and Rotation Forest algorithms for classifying prediction outcomes as severe or non-severe	Accuracy for: total UPDRS—76.09% motor UPDRS—79.49%
Mohammed et al., 2021 [4]	Voice UCI PD dataset	Feature selection and classification	Accuracy—96.6%
Velmurugan and Dhinakaran, 2022 [5]	UCI machine learning repository	Combination of linear regression and Adaboost ensemble methods with Random Forest (RF) and extreme gradient boosting (XGBoost)	Accuracy—90.13%
Sharma et al., 2021 [6]	Dataset collected by Max Little of Oxford University for voice disorders by collaborating with the National Centre for Voice and Speech	Feature selection using the Rao algorithm and classification using the k-Nearest Neighbors (KNN) classifier	Accuracy—99.25%
Sabeena et al., 2022 [7]	Voice dataset from the UCI machine learning repository	Feature selection using optimization-based ensembles and different classification algorithms	Accuracy ranging from 83.66% to 98.77%.
Ul Haq et al., 2020 [8]	Voice dataset	Feature selection using different feature selection methods and different classification using Support Vector Machine (SVM)	Accuracy ranging from 98.20% to 99.50%
Sarankumar et al., 2022 [9]	Voice dataset	Feature selection using the firming bacteria foraging algorithm and classification using the Deep Brooke inception net algorithm	Accuracy—99.88%
Pahuja and Nagabhushan, 2021 [10]	Voice dataset from the UCI repository	Classification using Artificial Neural Network (ANN), Support Vector Machine (SVM) and k-Nearest Neighbors (KNN) algorithms	Accuracy—95.89%, 88.21%, and 72.31%, respectively
Yücelbaş, 2021 [11]	Voice dataset	Feature selection using the Information gain algorithm-based KNN hybrid model (IGKNN)	Accuracy—98%
Pramanik et al., 2021 [12]	Acoustic features from the UCI machine learning repository	Feature selection using Correlated Feature Selection (CFS), Fisher Score Feature Selection (FSFS), and Mutual Information-based Feature Selection (MIFS) techniques, and classification using Naïve Bayes classifier	Accuracy—78.97%
Salmanpour, Shamsaei, Saberi, et al., 2021 [13]	Combination of non-imaging, imaging, and radiomic features from DAT-SPECT images	Sixteen algorithms for feature reduction, eight algorithms for clustering, and 16 classifiers	Subdivided the PD into three subtypes, namely mild, intermediate, and severe
Nahar et al., 2021 [14]	Acoustic features from the UCI machine learning repository	Feature selection using Boruta, Recursive Feature Elimination (RFE), and Random Forest (RF), and classification using Gradient Boosting, Extreme Gradient Boosting, Bagging, and Extra Tree Classifier	Accuracy—82.35% from applying the RFE feature selection methods and Bagging classifier

**Table 2:** List of subjects with sex, age, Parkinson’s stage, and number of years since diagnosis ^1^. Entries labeled “n/a” for healthy subjects for whom Parkinson’s stage and years since diagnosis are not applicable. “H&Y” refers to the Hoehn and Yahr PD stage, where higher values indicate a greater level of disability.

Subject Code	Sex	Age	Stage (H&Y)	Years Since Diagnosis
S01	M	78	3.0	0
S34	F	79	2.5	¼
S44	M	67	1.5	1
S20	M	70	3.0	1
S24	M	73	2.5	1
S26	F	53	2.0	1½
S08	F	48	2.0	2
S39	M	64	2.0	2
S33	M	68	2.0	3
S32	M	50	1.0	4
S02	M	60	2.0	4
S22	M	60	1.5	4½
S37	M	76	1.0	5
S21	F	81	1.5	5
S04	M	70	2.5	5½
S19	M	73	1.0	7
S35	F	85	4.0	7
S05	F	72	3.0	8
S18	M	61	2.5	11
S16	M	62	2.5	14
S27	M	72	2.5	15
S25	M	74	3.0	23
S06	F	63	2.5	28
S10 (healthy)	F	46	n/a	n/a
S07 (healthy)	F	48	n/a	n/a
S13 (healthy)	M	61	n/a	n/a
S43 (healthy)	M	62	n/a	n/a
S17 (healthy)	F	64	n/a	n/a
S42 (healthy)	F	66	n/a	n/a
S50 (healthy)	F	66	n/a	n/a
S49 (healthy)	M	69	n/a	n/a

^1^ Adapted from [15].

**Table 3:** Results on applying filter feature selection.

Classification Model	Accuracy (in %) after Filter Feature Selection	
	Dataset 1	Dataset 2
GuassianNB	67.34	75.13
SVM	85.71	76.72
Decision Tree-entropy	100.00	76.72
Random Forest	97.95	92.06
XGboost	100.00	86.24

**Table 4:** Results on applying ensemble learning methods to Dataset 1.

Ensemble Learning Method		Accuracy (in %)
Voting	Hard voting	100.00
	Soft voting	100.00
Stacking	Stacking	96.20
Bagging	Decision Tree-entropy	91.05
	Random Forest	89.70
	XGBoost	92.40

**Table 5:** Results on applying ensemble learning methods to Dataset 2.

Ensemble Learning Method		Accuracy (in %)
Voting	Hard voting	91.53
	Soft voting	88.89
Stacking	Stacking	90.06
Bagging	Decision Tree-entropy	88.34
	Random Forest	86.56
	XGBoost	87.76

**Table 6:** Results from applying genetic selection.

Classification Model	Accuracy (in %) after Filter Feature Selection	
	Dataset 1	Dataset 2
GuassianNB	91.83	77.63
SVM	81.63	77.63
Decision Tree-entropy	81.63	76.65
Decision Tree-Gini	81.63	74.34
Random Forest	83.67	74.34
XGboost	87.75	76.97
Logistic Regression	89.79	76.97

**Table 7:** Precision in applying filter feature selection.

Classification Model	Precision (in %) after Filter Feature Selection			
	Dataset 1		Dataset 2	
	PD Patient	Healthy	PD Patient	Healthy
GuassianNB	100	41	83	48
SVM	84	100	77	100
Decision Tree-entropy	100	100	86	51
Random Forest	100	92	94	86
XGboost	100	100	88	79

**Table 8:** Precision in applying genetic algorithms.

Classification Model	Precision (in %) after Genetic Selection			
	Dataset 1		Dataset 2	
	PD Patient	Healthy	PD Patient	Healthy
GuassianNB	90	100	78	73
SVM	85	62	78	78
Decision Tree-entropy	84	67	76	62
Decision Tree-Gini	85	62	77	50
Random Forest	89	64	74	0
XGBoost	94	69	78	64
Logistic Regression	92	80	79	61

## Data Availability

The datasets are available online in [15,16].
